# Advances in Viral Vector-Based TRAIL Gene Therapy for Cancer

**DOI:** 10.3390/cancers3010603

**Published:** 2011-02-10

**Authors:** Lyse A. Norian, Britnie R. James, Thomas S. Griffith

**Affiliations:** 1 Department of Urology, University of Iowa Carver College of Medicine, Iowa City, IA 52242, USA; E-Mail: lyse-norian@uiowa.edu; 2 Interdisciplinary Graduate Program in Immunology, University of Iowa Carver College of Medicine, Iowa City, IA 52242, USA; E-Mail: britnie-spaunhorst@uiowa.edu

**Keywords:** TRAIL, gene therapy, viral vectors, cancer, apoptosis

## Abstract

Numerous biologic approaches are being investigated as anti-cancer therapies in an attempt to induce tumor regression while circumventing the toxic side effects associated with standard chemo- or radiotherapies. Among these, tumor necrosis factor-related apoptosis-inducing ligand (TRAIL) has shown particular promise in pre-clinical and early clinical trials, due to its preferential ability to induce apoptotic cell death in cancer cells and its minimal toxicity. One limitation of TRAIL use is the fact that many tumor types display an inherent resistance to TRAIL-induced apoptosis. To circumvent this problem, researchers have explored a number of strategies to optimize TRAIL delivery and to improve its efficacy via co-administration with other anti-cancer agents. In this review, we will focus on TRAIL-based gene therapy approaches for the treatment of malignancies. We will discuss the main viral vectors that are being used for TRAIL gene therapy and the strategies that are currently being attempted to improve the efficacy of TRAIL as an anti-cancer therapeutic.

## 1. Introduction: TRAIL as an Anti-cancer Biologic Agent

Vast amounts of time and money have been spent developing new, more efficacious treatment options for cancer patients. Despite the many significant medical breakthroughs that have been made in recent years, advanced cancers remain difficult to treat, and standard therapies such as chemotherapy and radiotherapy are often associated with substantial toxicities that can limit their use in the clinic. For these reasons, biologic therapies have been increasingly explored as alternative approaches due to their potential to specifically target tumor cells for eradication, while leaving untransformed cells intact. By restricting cell death to only malignant cells, many of the side effects associated with standard therapies can be potentially avoided.

TNF-related apoptosis-inducing ligand (TRAIL) is a biologic therapy that has shown great promise in pre-clinical studies, due to its ability to preferentially induce apoptotic cell death in transformed cells. Apoptosis is a tightly regulated cellular process, and differs from necrosis in that it does not trigger inflammatory host responses. Apoptosis proceeds by two main pathways, referred to as intrinsic, which is controlled by interactions of pro-apoptotic and anti-apoptotic members of the B-cell leukemia/lymphoma 2 (Bcl-2) protein family [1], and extrinsic, which is induced via ligation of apoptosis-inducing “death receptors” on the cell surface [2]. Malignant cells often carry mutations in the proteins that control intrinsic and extrinsic apoptotic signaling pathways, resulting in a decreased susceptibility to cell death. Consequently, many tumor types have shown a resistance to TRAIL-induced cell death, thereby reducing its efficacy as a single agent [2-4]. To circumvent this deficiency, many groups have explored the use of TRAIL-based combinatorial approaches to cancer treatment, with the goal of overriding specific checkpoints that limit the clinical application of TRAIL “standalone” therapies.

Therapeutic TRAIL use has been investigated in many different formats. Initial approaches employed systemic or local administration of recombinant TRAIL protein to ligate the TRAIL receptors on cancer cells and activate the extrinsic cell death machinery [5]. As the cell surface receptors for TRAIL were identified, antibody-based therapies were used to specifically bind the death-inducing TRAIL receptors on cancer cells and trigger apoptosis [5-7]. A number of recent reviews have covered these approaches [4,8], so we will instead focus on gene therapy-based methodologies that stimulate the TRAIL/TRAIL receptor cell death pathway in tumor cells. Within this context, we will summarize the TRAIL receptor family, the intracellular pathways that culminate in apoptosis following ligation of those receptors, the approaches that have been undertaken to enhance tumor cell susceptibility to TRAIL-mediated cell death, and the viral vectors that are being used to induce TRAIL gene expression.

## 2. Structure and Function of TRAIL

Prior to the discovery of TRAIL, two other members of the TNF family—the prototypic TNF and the closely related Fas Ligand (FasL)—had been identified as potent inducers of tumor cell death. Unfortunately, significant toxicity (*i.e.*, induction of normal cell/tissue death) was observed after systemic administration of TNF and FasL (or anti-Fas mAb), which put the utility of these molecules as anti-cancer therapeutics into question. In 1995, Wiley *et al.* reported the cloning of the full length TRAIL cDNA [9]. A few months later, Pitti *et al.* published a report describing the same protein, which they called Apo-2 ligand [10]. These two reports defined TRAIL as a Type II transmembrane protein that can be released as a soluble form following cleavage of the C-terminal extracellular domain. Evaluation of the extracellular domain of TRAIL revealed a moderate degree of homology with other TNF family members, including FasL (28% amino acid identity), TNF (23%), lymphotoxin-α (23%), and lymphotoxin-β (22%). Although the amino acid homology between TRAIL and these proteins was modest, the three-dimensional crystal structure of TRAIL was found to be quite similar to that of TNF and CD40 ligand [11]. TRAIL monomers are made up of two anti-parallel β-pleated sheets that form a β sandwich core; the monomers then interact in a head-to-tail fashion to form a bell-shaped trimer [11]. This is an important hallmark of numerous TNF family members, in that trimerization leads to much greater biologic activity than is observed for either the monomeric or dimeric forms [9]. In humans, TRAIL mRNA is present in a variety of tissues including the spleen, thymus, prostate, ovary, small intestine, colon and placenta [9]. Within the hematopoietic compartment, TRAIL is expressed on activated T lymphocytes, B cells, NK cells, monocytes, dendritic cells, and neutrophils [3,12-16].

Early *in vitro* studies on TRAIL function revealed that it preferentially induced apoptosis in transformed cells, while leaving normal cells and tissues intact [9], and multiple *in vivo* studies have confirmed that the primary targets of TRAIL-induced apoptosis are malignant cells [17-21]. Further investigation into TRAIL function in tumor-free model systems has demonstrated that it can, in fact, induce apoptosis in specific populations of untransformed cells such as activated lymphocytes and influenza-infected epithelial cells of the lung [22-24]. So, depending on the pathophysiological condition, the apoptosis-inducing ability of TRAIL may not be as selective for transformed cells as was originally believed. Despite the fact that noncancerous cells can also be killed by TRAIL under certain circumstances, the development of TRAIL as an anti-cancer agent has continued and the vast majority of work with TRAIL has focused on its tumoricidal activity.

## 3. The TRAIL Receptor Family

TRAIL (either soluble or membrane-bound) can bind to one of several receptors. In humans, four membrane-bound TRAIL receptors have been identified: TRAIL receptor-1 [Death Receptor 4 (DR4)] [25], TRAIL receptor-2 [Death Receptor 5 (DR5)] [26,27], TRAIL receptor-3 [Decoy Receptor 1 (DcR1)/TRAIL receptor without an intracellular domain (TRID)] [26,28,29], and TRAIL receptor-4 [Decoy Receptor 2 (DcR2)/TRAIL receptor with a truncated death domain (TRUNDD)] [29-32]. The cytoplasmic tails of TRAIL-R1 and -R2 contain functional death domains that transduce apoptotic signals after receptor trimerization [32]. In contrast, neither TRAIL-R3 nor -R4 possess a functional intracellular death domain, and consequently have been referred to as “decoy receptors” because they bind TRAIL with similar affinity as TRAIL-R1 and -R2 but cannot initiate apoptotic signaling. TRAIL-R3 and -R4 can therefore be considered natural competitive inhibitors that, when expressed at the right ratio with TRAIL-R1 and/or -R2, diminish the net apoptotic activity of TRAIL protein [28]. TRAIL can also bind to the soluble TNF receptor superfamily member osteoprotegerin [33], which functions primarily as a decoy receptor for Receptor Activator of NF-κB Ligand (RANKL) [34]. In mice, TRAIL is thought to bind to three receptors: TRAIL-R/DR5, mDcTRAIL-R1, and mDcTRAIL-R2. Of these murine TRAIL receptors, only DR5 has been characterized at the functional level [35,36].

## 4. Signaling Cascades that Regulate TRAIL-Induced Apoptosis

**Figure 1.**
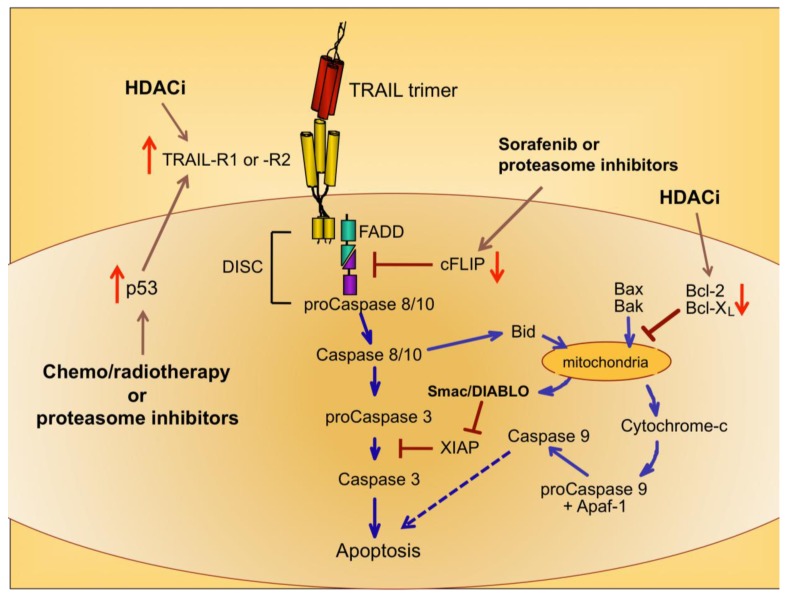
Therapeutic intervention points in the signaling pathways that mediate TRAIL-induced apoptosis. Intracellular pathways that culminate in apoptosis following TRAIL-R1 and -R2 ligation are illustrated. Blue arrows indicate apoptotic signaling pathways. Brown arrows indicate molecular intervention points for therapies that augment TRAIL-induced apoptosis. Red arrows indicate up- or down-regulation of specific molecular targets.

Upon ligation of TRAIL trimers to TRAIL-R1 or -R2 in humans, the extrinsic apoptotic pathway is activated via a series of intracellular changes that culminate in the apoptotic death of the TRAIL receptor-bearing cell (Figure 1). One of the most proximal events of the TRAIL-induced signaling cascade is formation of a multimeric protein structure called the death-inducing signaling complex (DISC). The functional DISC is comprised of several proteins, including the ligated death receptors, Fas-associated death domain protein (FADD), and procaspases 8 and 10 [37,38]. Following DISC formation, the downstream signaling cascade is initiated via autocatalytic cleavage of procaspase 8 into its active form [38]. Active caspase 8 then amplifies the apoptotic signal by cleaving and activating the effector caspases 3, 6, and 7. Caspases 3, 6, and 7 actively promote cell death by cleaving multiple protein targets that are responsible for maintaining cellular integrity, resulting in the cellular hallmarks of apoptosis: plasma membrane blebbing, inter-nucleosomal DNA cleavage, and nuclear shrinking. Active caspase 8 can also cleave the pro-apoptotic Bcl-2 protein Bid, thereby simultaneously triggering the intrinsic apoptotic pathway [39,40]. Transduction of pro-apoptotic signaling from the DISC is tightly regulated, and cellular FADD-like IL-1β-converting enzyme inhibitory protein (cFLIP) is a critical inhibitor at this proximal point in the apoptosis signal transduction pathway [41].

Cells expressing functional TRAIL receptors can be classified as either Type I or Type II, depending on their differential requirements for involvement of the intrinsic pathway in triggering apoptosis. Type I cells undergo apoptosis in response to extrinsic signals that lead to caspase 8 cleavage. Type II cells do not undergo apoptosis upon activation of the extrinsic pathway alone; they also require activation of the intrinsic apoptotic pathway and inactivation of intracellular proteins that inhibit caspase signaling cascades, such as X-linked Inhibitor of Apoptosis Protein (XIAP) [42]. Initiation of the intrinsic apoptotic pathway leads to a loss of mitochondrial membrane potential. This allows cytochrome c to escape from the mitochondria into the cytosol, and also releases Second Mitochondria-derived Activator of Caspases/Direct Inhibitor of Apoptosis Protein Binding Protein with Low Isoelectric Point (Smac/DIABLO), which blocks the function of caspase inhibitors such as XIAP (Figure 1) [43,44]. Cytosolic cytochrome c promotes apoptosis by contributing to the formation of the apoptosome, which also includes ATP and Apoptotic Peptidase-Activating Factor 1 (APAF-1), and results in activation of caspase 9 [45,46]. Thus, co-induction of the intrinsic apoptotic pathway results in greater overall caspase activity, and a stronger pro-apoptotic signal that robustly promotes cell death.

## 5. Overcoming Tumor Cell Resistance to TRAIL-Induced Apoptosis

The focus on TRAIL as an anti-cancer agent originated from early studies that showed its ability to induce apoptosis was largely restricted to malignant cells [9]. In one report, 32 of 39 human tumor cell lines were sensitive to TRAIL-induced apoptosis *in vitro*, whereas untransformed cells were resistant [19]. This same study demonstrated that repeated intravenous injections of human TRAIL caused no observable toxicity in non-human primates [19]. Since that time, numerous reports have demonstrated the preferential ability of TRAIL to induce apoptosis in tumor cells, both *in vitro* and *in vivo*, while producing minimal toxic side effects in hosts [9,19-21,47-49]. Despite these encouraging results, other reports have shown that many tumor types are resistant to TRAIL (reviewed in [2,4,5]). To circumvent this limitation, a wide array of combinatorial therapies based on TRAIL administration has been examined, and several of these are outlined below.

### 5.1. Chemotherapy and Radiotherapy

Because chemo- and radiotherapy are the standard of care for many malignancies, their ability to synergize with TRAIL administration has been investigated. These therapeutic approaches tend to induce the tumor suppressor p53, which normally accumulates in cells following DNA damage [50]. Increased p53 expression can lead to increased TRAIL-R1 and –R2 expression [51,52], providing a mechanism for heightened TRAIL sensitivity (Figure 1) [53,54]. Therefore, combining TRAIL administration with standard chemo- or radiotherapy may prove more efficacious than administering TRAIL alone, as suggested by promising results in pre-clinical models of glioma, renal cell carcinoma, breast, prostate, and bladder cancer [55-59].

### 5.2. Proteasome Inhibitors

A variety of drugs can increase tumor cell sensitivity to TRAIL-mediated apoptosis, usually by bypassing the intracellular checkpoints that render cells resistant to TRAIL. For example, the proteasome inhibitor bortezomib, approved for the treatment of multiple myeloma, promotes apoptosis by preventing ubiquitin-mediated degradation of pro-apoptotic proteins, increasing p53 and TRAIL-R2 expression, and decreasing cFLIP expression (Figure 1) [60-62]. A recent report examining the combined effects of bortezomib and TRAIL on 15 different squamous cell carcinoma lines found that bortezomib also enhanced TRAIL efficacy through increased recruitment of caspase 8 and FADD into the DISC, and augmentation of the intrinsic apoptotic pathway [63]. For these reasons, the combination of TRAIL and bortezomib has shown promise as a therapeutic for TRAIL-resistant breast, colon and kidney tumors [64].

### 5.3. Sorafenib

Sorafenib is a protein tyrosine kinase inhibitor approved for use in the treatment of renal cell carcinoma, and it has also received attention for its ability to sensitize tumor cells to TRAIL-mediated apoptosis by shifting the intracellular ratio of pro-apoptotic and anti-apoptotic molecules [65]. This can occur through several mechanisms, including sorafenib-triggered proteolytic degradation of cFLIP (Figure 1) [66] and decreased expression of the anti-apoptotic Bcl-2 family member Mcl-1 [67].

### 5.4. Histone Deacetylase Inhibitors

Histone deacetylase inhibitors (HDACi) are a new class of antitumor drugs that epigenetically alter gene expression through increased histone acetylation [68], which in turn leads to the transcription of genes, such as tumor suppressors, that are normally repressed during tumor outgrowth. HDACi are being investigated as stand-alone agents for a variety of cancers, but they are also being explored for their ability to sensitize tumor cells to TRAIL-mediated killing. HDACi upregulate TRAIL-R2 expression on tumor cells, thereby leading to a greater susceptibility to TRAIL-induced apoptosis (Figure 1) [69-73]. In addition, HDAC inhibition can modulate TRAIL-induced apoptosis in tumor cells by other molecular mechanisms, including increased caspase activation and decreased expression of anti-apoptotic molecules such as Bcl-2 [71,74,75].

## 6. Viral Vector-Mediated Gene Therapy

Depending on the disease, gene therapy has become a viable therapeutic alternative for patients over the last decade because this technology can be used to restore proper gene expression in host cells, to express proteins with the intent of stimulating immunity (*i.e.* immunization), and/or to introduce genes that encode cytotoxic proteins. There has also been a great deal of work performed in the gene therapy arena focused on improving the delivery of therapeutic genes by modifying viral vectors to increase their tropism for target cells, or by restricting transgene expression via incorporation of cell/tissue-specific promoters. Regardless of its intended use, one of the most important aspects of viral vector-based gene therapy is that this type of treatment should be capable of treating the disease in question while remaining benign to the host.

Viruses act as natural vehicles for delivering genes as they readily transfer their genetic material to infected host cells [76,77]. For use as therapeutic agents, viral vectors are frequently genetically modified to circumvent productive infection and toxicity, while still allowing for expression of virally-derived proteins in host cells. A number of viral vectors have been described, from both RNA and DNA genome origins, and each has unique advantages in terms of the types of cells that can be infected and the ability to express transgenes either transiently or permanently. Among the most frequently studied viral vectors are adenovirus (Ad) and adeno-associated virus (AAV) [76-78]. Ad- and AAV-based vectors have been used in a number of pre-clinical studies examining the potential of viral vector-mediated TRAIL administration via gene therapy. Here we will discuss the properties that make both Ad and AAV desirable candidates for TRAIL-based gene therapy.

### 6.1. Adenovirus

Discovered in 1953, Ad is a non-enveloped virus with a double stranded 26-45 kb DNA genome [79]. There are 51 identified human Ad serotypes, with types 2 (Ad2) and 5 (Ad5) being the best characterized [76,77]. Ad2 and Ad5 have high tropism for numerous cell types, do not cause extensive disease in humans, and were the first established vectors for gene therapy [80,81]. Because Ad do not integrate into the host genome, the transgenes are transiently expressed [76]. The transient gene expression exhibited with Ad vectors favors gene therapy situations where permanent transgene expression is not required.

Ad vectors can be engineered to remain replication-sufficient, conditionally replication-sufficient, or replication-deficient in the host. First-generation Ad vectors contained a deletion of the early gene 1 (*E1A*), which is activated upon entry into the host cell and vital for transcription and function of the other early genes [82]. Deletion of *E1A* also renders Ad replication-deficient and allows space within the genome for transgene insertion. *E3* deletion has no apparent effect on viral infectivity [83], and in most first-generation Ad5 vectors, both *E1A* and *E3* were deleted to create a replication-deficient vector that could accommodate larger transgene insertions [83]. Many second-generation Ad5 vectors have also had the *E2* region deleted to alleviate host inflammatory responses and potential toxicity [84].

For entry into a cell by receptor-mediated endocytosis, Group C adenoviruses (e.g. Ad2 and Ad5) require interaction between the viral fiber capsid protein with the coxsackievirus and adenovirus receptor (CAR), and the viral penton base binding to α_v_ integrins [85-87]. Thus, the success of these Ad-based therapies is primarily dictated by CAR recognition [88]. CAR is ubiquitously expressed in most benign epithelial tissues, yet marked variations in CAR levels have been demonstrated using different cancer cell lines of the same tissue origin [89]. Cellular resistance to Ad infection can, therefore, further complicate (and potentially limit) the utility of Ad-mediated gene delivery.

Modifications to the Ad vector can aid in overcoming cellular resistance. A modification to the viral fiber, via insertion of an Arg-Gly-Asp (RGD) integrin-binding motif, allows for CAR-independent binding interactions [86,89]. Fiber-modified Ad vectors encoding TRAIL have been used to induce apoptosis of human pancreatic cancer *in vitro* and *in vivo* [90] and colon cancer *in vitro* [91]. Importantly, the modified Ad-TRAIL-RGD vector also elicited cytotoxic effects in adenovirus-resistant tumors and cancer cells with low to negative CAR expression [92]. Similarly, Ad5 vectors that co-express Group B-derived Ad35 capsid fibers can recognize CD46 instead of CAR, which increases the tropism of the vector for tumor cells [93,94]. An Ad5/35 vector encoding TRAIL exhibited enhanced tumor cell killing in a model of human glioblastoma *in vitro* and *in vivo* [94]. Alternatively, tumor cell treatment with HDACi can result in increased CAR and α_v_ integrin expression, which enhances adenoviral infection and transgene expression [72].

Site-specific cell targeting for directed transgene expression is another modification that can be made in Ad vectors. For example, targeting Ad-mediated transgene delivery to tumor cells has been achieved through expression of the transgene under the human telomerase reverse transcriptase (hTERT) promoter [95]. Telomerase activity can be used as a marker for tumor cell activity, so expression of TRAIL under the hTERT promoter allows for site-specific expression of TRAIL [95]. Tissue specificity can also be achieved using tissue specific promoters, such as prostate-specific enhancing sequences (PSES). The addition of this promoter was found to limit TRAIL expression to prostate-specific antigen (PSA)- and prostate-specific membrane antigen (PSMA)-expressing cells [96]. Thus, the refinement of Ad vectors for TRAIL-based gene therapy continues, with the goal of increasing TRAIL transgene expression specifically in transformed tumor cells, thereby increasing the potential therapeutic benefits of this approach while simultaneously minimizing toxicity.

We were the first to describe the *in vitro* and *in vivo* tumoricidal activity of Ad-TRAIL [97,98]. These initial studies were designed to only examine Ad-TRAIL-induced tumor cell death, and a number of other investigators have evaluated similar TRAIL-encoding Ad vectors in a variety of tumor models [21,91,99]. Our more recent studies have examined the impact of Ad-TRAIL-induced tumor cell death on the subsequent induction of systemic anti-tumor immunity. Using a murine model of RCC, we demonstrated that combinatorial therapy with Ad-TRAIL and CpG-containing oligonucleotides increased tumor regression and prolonged animal survival [100]. Data from this report also showed that Ad-TRAIL/CpG therapy led to the generation of immunological memory, since mice that went on to clear the primary tumor after treatment were also able to resist a second tumor challenge. These preclinical results suggesting the therapeutic potential of Ad-TRAIL gave us the necessary “proof-of-concept” data to justify initiation of a phase I clinical trial in men with prostate cancer. Our preliminary results from that trial showed that intra-prostatic injection of Ad-TRAIL was well-tolerated in patients, and produced no adverse side effects [5]. In addition, there was evidence of apoptotic death (DNA fragmentation via TUNEL staining), which suggested that TRAIL expressed from the transferred transgene was functional. Unfortunately, this phase I trial was not designed to test therapeutic efficacy. Thus, it will be interesting to continue our evaluation of Ad-TRAIL clinically.

### 6.2. Adeno-Associated Virus

In addition to Ad vectors for gene therapy, AAV vectors have also been engineered and tested as gene transfer vectors. AAV is a small, nonenveloped, single stranded DNA virus [101]. Unlike Ad, only six AAV serotypes have been identified in primates, with AAV-2 being the most extensively studied and used in gene therapy. AAV has not been conclusively shown to cause human disease, and elicit minimal cellular immune responses in the host, with little inflammation and cellular activation. AAV infection is essentially non-productive without the presence of a helper virus, usually Ad. Consequently, the generation of AAV vectors is more difficult than other recombinant viral vectors because AAV vectors require co-transfection with helper protein plasmids, and low viral titers are frequently obtained [78,102].

However, AAV vectors are desirable for, and have been successfully used for, gene therapy due to their other unique features. For example, AAV has high tropism for many proliferating and quiescent cell types, and in human cells AAV is able to site-specifically integrate into chromosome 19. Despite chromosome integration, AAV transgene expression is not life-long in the host; however, expression does have the potential to persist for several years *in vivo* [78,101,102]. AAV can also be found extrachromosomally as an episomal AAV genome, which decreases the risk of random integration (another feature that makes it desirable as a gene therapy vector). Due to the small size of AAV, only transgene inserts smaller than 5 kb can be inserted. Interestingly, AAV has the ability to heterodimerize, which allows genes to be “split” between two vectors, resulting in expression of an intact transgene only after post-transcriptional modifications occur [78]. Modifications can be made to the AAV vector to increase tropism, enhance transduction and to evade the immune response [103]. Also, mosaic vectors consisting of components from multiple AAV can be used to allow for tissue-specific targeting and to broaden cell/tissue tropism. Together, these unique features of AAV have made it a successful vector in pre-clinical research for gene therapy.

The positive features of AAV have made it a popular vector option for TRAIL gene administration. In a model of colorectal cancer, AAV-encoded expression of TRAIL resulted in rapid transgene expression, followed by apoptosis of several tumor cell lines and either complete eradication of tumors or blunted progression of existing tumors [104]. Similar results were seen for an AAV vector encoding soluble TRAIL, using A549 established tumors in nude mice [105]. Mosaic AAV vectors contain a mixture of capsid proteins from multiple AAV capable of binding different cellular receptors allowing for a broader cellular tropism [106]. Mosaic vector, AAV2/5, consists of both serotypes 2, which binds hepran sulfate proteoglycans, and 5, which binds sialic acid. Shi e*t al.* utilized AAV2/5 encoding TRAIL and showed increased tropism for the A549 cell line *in vitro* and *in vivo*, while histology and primary cell culture showed no detectable toxicity to normal tissues [107]. Similar to Ad, tissue- and tumor-specific targeting of the AAV vector can be established with insertion of target sequences [95,108]. Tumor cell-specific AAV vectors have been engineered with TRAIL expression under the control of the telomerase reverse transcriptase (hTERT) promoter [95]. Treatment of human hepatocellular carcinoma cells with AAV-hTERT-TRAIL demonstrated cancer-specific cytoxicity [109,110]. *In vivo* experiments exhibited hepatocellular specific tumor cell death and tumor regression when treated with AAV-hTERT-TRAIL [109-111]. Tissue-targeting AAV vectors have also been engineered for site-specific TRAIL expression. Ma *et al.* achieved hepatocellular-specific TRAIL expression by fusing the human insulin signal peptide to TRAIL (AAV-ISN-T) within the vector [112]. Oral administration of AAV-ISN-T to mice bearing subcutaneous SMMC-7721 (human liver cancer) tumors produced long-lasting expression of soluble TRAIL in the liver, which resulted in tumor regression [112].

### 6.3. Other Vector Options

With low cytoxicity in normal tissues and site-specific therapeutic effects, treatment with Ad- or AAV-vector encoded TRAIL in pre-clinical studies has provided strong evidence for future gene therapy of cancer patients. While TRAIL-encoding Ad and AAV have been the most popular vectors so far, other TRAIL-encoding vectors have been investigated. Lentiviral-mediated TRAIL expression was shown to be successful for specific induction of apoptosis in human lung cancer cells *in vitro*, but was not as efficacious *in vivo* [113]. A genetically engineered synthetic biomimetic vector has also been used to co-administer plasmid DNA specific for TRAIL. Transfection of ZR-75-1 breast cancer cells with the biomimetic TRAIL vector demonstrated increased tumor cell killing [114], indicating that this approach warrants additional investigation.

## 7. Conclusions

In summary, the use of TRAIL-based gene therapy for cancer patients holds a great deal of promise. Known limitations of TRAIL, such as resistance to TRAIL-mediated killing, and cancer cell resistance to viral vector infection are being overcome in a variety of ways. Pre-clinical studies in mice have demonstrated the safety and efficacy of viral-based gene therapy using TRAIL, and phase I clinical trial results have demonstrated the safety of this approach. These advances pave the way for future clinical applications of TRAIL gene therapy as a treatment option for a variety of malignancies.

## References

[1] Wei M.C., Zong W.X., Cheng E.H., Lindsten T., Panoutsakopoulou V., Ross A.J., Roth K.A., MacGregor G.R., Thompson C.B., Korsmeyer S.J. (2001). Proapoptotic Bax and Bak: A requisite gateway to mitochondrial dysfunction and death. Science.

[2] Testa U. (2010). Trail/trail-r in hematologic malignancies. J. Cell. Biochem..

[3] Zamai L., Ahmad M., Bennett I.M., Azzoni L., Alnemri E.S., Perussia B. (1998). Natural killer (NK) cell-mediated cytotoxicity: Differential use of TRAIL and Fas ligand by immature and mature primary human NK cells. J. Exp. Med..

[4] Mellier G., Huang S., Shenoy K., Pervaiz S. (2010). Trailing death in cancer. Mol. Aspects Med..

[5] Holoch P.A., Griffith T.S. (2009). TNF-related apoptosis-inducing ligand (TRAIL): A new path to anti-cancer therapies. Eur. J. Pharmacol..

[6] Rosevear H.M., Lightfoot A.J., Griffith T.S. (2010). Conatumumab, a fully human mAb against death receptor 5 for the treatment of cancer. Curr. Opin. Investig. Drugs.

[7] Griffith T.S., Rauch C.T., Smolak P.J., Waugh J.Y., Boiani N., Lynch D.H., Smith C.A., Goodwin R.G., Kubin M.Z. (1999). Functional analysis of TRAIL receptors using monoclonal antibodies. J. Immunol..

[8] Wiezorek J., Holland P., Graves J. (2010). Death receptor agonists as a targeted therapy for cancer. Clin. Cancer Res..

[9] Wiley S.R., Schooley K., Smolak P.J., Din W.S., Huang C.P., Nicholl J.K., Sutherland G.R., Smith T.D., Rauch C., Smith C.A. (1995). Identification and characterization of a new member of the TNF family that induces apoptosis. Immunity.

[10] Pitti R.M., Marsters S.A., Ruppert S., Donahue C.J., Moore A., Ashkenazi A. (1996). Induction of apoptosis by Apo-2 ligand, a new member of the tumor necrosis factor cytokine family. J. Biol. Chem..

[11] Cha S.S., Kim M.S., Choi Y.H., Sung B.J., Shin N.K., Shin H.C., Sung Y.C., Oh B.H. (1999). 2.8 A resolution crystal structure of human TRAIL, a cytokine with selective antitumor activity. Immunity.

[12] Griffith T.S., Wiley S.R., Kubin M.Z., Sedger L.M., Maliszewski C.R., Fanger N.A. (1999). Monocyte-mediated tumoricidal activity via the tumor necrosis factor-related cytokine, TRAIL. J. Exp. Med..

[13] Fanger N.A., Maliszewski C.R., Schooley K., Griffith T.S. (1999). Human dendritic cells mediate cellular apoptosis via tumor necrosis factor-related apoptosis-inducing ligand (TRAIL). J. Exp. Med..

[14] Kemp T.J., Moore J.M., Griffith T.S. (2004). Human B cells express functional TRAIL/Apo-2 ligand after CpG-containing oligodeoxynucleotide stimulation. J. Immunol..

[15] Kemp T.J., Ludwig A.T., Earel J.K., Moore J.M., Vanoosten R.L., Moses B., Leidal K., Nauseef W.M., Griffith T.S. (2005). Neutrophil stimulation with Mycobacterium bovis bacillus calmette-guerin (BCG) results in the release of functional soluble TRAIL/Apo-2L. Blood.

[16] Simons M.P., Leidal K.G., Nauseef W.M., Griffith T.S. (2008). TNF-related apoptosis-inducing ligand (TRAIL) is expressed throughout myeloid development, resulting in a broad distribution among neutrophil granules. J. Leukoc. Biol..

[17] Yang F., Shi P., Xi X., Yi S., Li H., Sun Q., Sun M. (2006). Recombinant adenoviruses expressing TRAIL demonstrate antitumor effects on non-small cell lung cancer (NSCLC). Med. Oncol..

[18] Walczak H., Miller R.E., Ariail K., Gliniak B., Griffith T.S., Kubin M., Chin W., Jones J., Woodward A., Le T., Smith C., Smolak P., Goodwin R.G., Rauch C.T., Schuh J.C., Lynch D.H. (1999). Tumoricidal activity of tumor necrosis factor-related apoptosis-inducing ligand *in vivo*. Nat. Med..

[19] Ashkenazi A., Pai R.C., Fong S., Leung S., Lawrence D.A., Marsters S.A., Blackie C., Chang L., McMurtrey A.E., Hebert A., DeForge L., Koumenis I.L., Lewis D., Harris L., Bussiere J., Koeppen H., Shahrokh Z., Schwall R.H. (1999). Safety and antitumor activity of recombinant soluble Apo2 ligand. J. Clin. Invest..

[20] Lin T., Zhang L., Davis J., Gu J., Nishizaki M., Ji L., Roth J.A., Xiong M., Fang B. (2003). Combination of TRAIL gene therapy and chemotherapy enhances antitumor and antimetastasis effects in chemosensitive and chemoresistant breast cancers. Mol. Ther..

[21] Huang X., Lin T., Gu J., Zhang L., Roth J.A., Stephens L.C., Yu Y., Liu J., Fang B. (2002). Combined TRAIL and Bax gene therapy prolonged survival in mice with ovarian cancer xenograft. Gene Ther..

[22] Gurung P., Kucaba T.A., Schoenberger S.P., Ferguson T.A., Griffith T.S. (2010). TRAIL-expressing CD8+ T cells mediate tolerance following soluble peptide-induced peripheral T cell deletion. J. Leukoc. Biol..

[23] Brincks E.L., Katewa A., Kucaba T.A., Griffith T.S., Legge K.L. (2008). CD8 T cells utilize TRAIL to control influenza virus infection. J. Immunol..

[24] Janssen E.M., Droin N.M., Lemmens E.E., Pinkoski M.J., Bensinger S.J., Ehst B.D., Griffith T.S., Green D.R., Schoenberger S.P. (2005). CD4+ T-cell help controls CD8+ T-cell memory via TRAIL-mediated activation-induced cell death. Nature.

[25] Pan G., O'Rourke K., Chinnaiyan A.M., Gentz R., Ebner R., Ni J., Dixit V.M. (1997). The receptor for the cytotoxic ligand TRAIL. Science.

[26] Pan G., Ni J., Wei Y.F., Yu G., Gentz R., Dixit V.M. (1997). An antagonist decoy receptor and a death domain-containing receptor for TRAIL. Science.

[27] Walczak H., Degli-Esposti M.A., Johnson R.S., Smolak P.J., Waugh J.Y., Boiani N., Timour M.S., Gerhart M.J., Schooley K.A., Smith C.A., Goodwin R.G., Rauch C.T. (1997). TRAIL-R2: A novel apoptosis-mediating receptor for TRAIL. EMBO J..

[28] Sheridan J.P., Marsters S.A., Pitti R.M., Gurney A., Skubatch M., Baldwin D., Ramakrishnan L., Gray C.L., Baker K., Wood W.I., Goddard A.D., Godowski P., Ashkenazi A. (1997). Control of TRAIL-induced apoptosis by a family of signaling and decoy receptors. Science.

[29] Degli-Esposti M.A., Dougall W.C., Smolak P.J., Waugh J.Y., Smith C.A., Goodwin R.G. (1997). The novel receptor TRAIL-R4 induces NF-kappaB and protects against TRAIL-mediated apoptosis, yet retains an incomplete death domain. Immunity.

[30] Marsters S.A., Sheridan J.P., Pitti R.M., Huang A., Skubatch M., Baldwin D., Yuan J., Gurney A., Goddard A.D., Godowski P., Ashkenazi A. (1997). A novel receptor for Apo2L/TRAIL contains a truncated death domain. Curr. Biol..

[31] Pan G., Ni J., Yu G., Wei Y.F., Dixit V.M. (1998). TRUNDD, a new member of the TRAIL receptor family that antagonizes TRAIL signalling. FEBS Lett..

[32] Hymowitz S.G., Christinger H.W., Fuh G., Ultsch M., O'Connell M., Kelley R.F., Ashkenazi A., de Vos A.M. (1999). Triggering cell death: The crystal structure of Apo2L/TRAIL in a complex with Death Receptor 5. Mol. Cell.

[33] Emery J.G., McDonnell P., Burke M.B., Deen K.C., Lyn S., Silverman C., Dul E., Appelbaum E.R., Eichman C., DiPrinzio R., Dodds R.A., James I.E., Rosenberg M., Lee J.C., Young P.R. (1998). Osteoprotegerin is a receptor for the cytotoxic ligand TRAIL. J. Biol. Chem..

[34] Lacey D.L., Timms E., Tan H.L., Kelley M.J., Dunstan C.R., Burgess T., Elliott R., Colombero A., Elliott G., Scully S., Hsu H., Sullivan J., Hawkins N., Davy E., Capparelli C., Eli A., Qian Y.X., Kaufman S., Sarosi I., Shalhoub V., Senaldi G., Guo J., Delaney J., Boyle W.J. (1998). Osteoprotegerin ligand is a cytokine that regulates osteoclast differentiation and activation. Cell.

[35] Wu G.S., Burns T.F., Zhan Y., Alnemri E.S., El-Deiry W.S. (1999). Molecular cloning and functional analysis of the mouse homologue of the Killer/DR5 tumor necrosis factor-related apoptosis-inducing ligand (TRAIL) death receptor. Cancer Res..

[36] Schneider P., Olson D., Tardivel A., Browning B., Lugovskoy A., Gong D., Dobles M., Hertig S., Hofmann K., Van Vlijmen H., Hsu Y.M., Burkly L.C., Tschopp J., Zheng T.S. (2003). Identification of a new murine tumor necrosis factor receptor locus that contains two novel murine receptors for tumor necrosis factor-related apoptosis-inducing ligand (TRAIL). J. Biol. Chem..

[37] Sprick M.R., Weigand M.A., Rieser E., Rauch C.T., Juo P., Blenis J., Krammer P.H., Walczak H. (2000). FADD/MORT1 and caspase-8 are recruited to TRAIL receptors 1 and 2 and are essential for apoptosis mediated by TRAIL receptor 2. Immunity.

[38] Kischkel F.C., Lawrence D.A., Chuntharapai A., Schow P., Kim K.J., Ashkenazi A. (2000). Apo2L/TRAIL-dependent recruitment of endogenous FADD and caspase-8 to death receptors 4 and 5. Immunity.

[39] Li H., Zhu H., Xu C.J., Yuan J. (1998). Cleavage of Bid by caspase 8 mediates the mitochondrial damage in the Fas pathway of apoptosis. Cell.

[40] Griffith T.S., Chin W.A., Jackson G.C., Lynch D.H., Kubin M.Z. (1998). Intracellular regulation of TRAIL-induced apoptosis in human melanoma cells. J. Immunol..

[41] Irmler M., Thome M., Hahne M., Schneider P., Hofmann K., Steiner V., Bodmer J.L., Schroter M., Burns K., Mattmann C., Rimoldi D., French L.E., Tschopp J. (1997). Inhibition of death receptor signals by cellular FLIP. Nature.

[42] Barnhart B.C., Alappat E.C., Peter M.E. (2003). The CD95 type I/type II model. Semin. Immunol..

[43] Du C., Fang M., Li Y., Li L., Wang X. (2000). Smac, a mitochondrial protein that promotes cytochrome c-dependent caspase activation by eliminating IAP inhibition. Cell.

[44] Verhagen A.M., Ekert P.G., Pakusch M., Silke J., Connolly L.M., Reid G.E., Moritz R.L., Simpson R.J., Vaux D.L. (2000). Identification of DIABLO, a mammalian protein that promotes apoptosis by binding to and antagonizing IAP proteins. Cell.

[45] Schulze-Osthoff K., Ferrari D., Los M., Wesselborg S., Peter M.E. (1998). Apoptosis signaling by death receptors. Eur. J. Biochem..

[46] Li P., Nijhawan D., Budihardjo I., Srinivasula S.M., Ahmad M., Alnemri E.S., Wang X. (1997). Cytochrome c and dATP-dependent formation of APAF-1/caspase-9 complex initiates an apoptotic protease cascade. Cell.

[47] Kelley S.K., Harris L.A., Xie D., Deforge L., Totpal K., Bussiere J., Fox J.A. (2001). Preclinical studies to predict the disposition of Apo2L/tumor necrosis factor-related apoptosis-inducing ligand in humans: Characterization of *in vivo* efficacy, pharmacokinetics, and safety. J. Pharmacol. Exp. Ther..

[48] Pollack I.F., Erff M., Ashkenazi A. (2001). Direct stimulation of apoptotic signaling by soluble Apo2L/tumor necrosis factor-related apoptosis-inducing ligand leads to selective killing of glioma cells. Clin. Cancer Res..

[49] Gazitt Y. (1999). TRAIL is a potent inducer of apoptosis in myeloma cells derived from multiple myeloma patients and is not cytotoxic to hematopoietic stem cells. Leukemia.

[50] Kemp C.J., Sun S., Gurley K.E. (2001). p53 induction and apoptosis in response to radio- and chemotherapy *in vivo* is tumor-type-dependent. Cancer Res..

[51] Liu X., Yue P., Khuri F.R., Sun S.Y. (2004). p53 upregulates Death Receptor 4 expression through an intronic p53 binding site. Cancer Res..

[52] Takimoto R., El-Deiry W.S. (2000). Wild-type p53 transactivates the Killer/DR5 gene through an intronic sequence-specific DNA-binding site. Oncogene.

[53] Gliniak B., Le T. (1999). Tumor necrosis factor-related apoptosis-inducing ligand's antitumor activity *in vivo* is enhanced by the chemotherapeutic agent CPT-11. Cancer Res..

[54] Chinnaiyan A.M., Prasad U., Shankar S., Hamstra D.A., Shanaiah M., Chenevert T.L., Ross B.D., Rehemtulla A. (2000). Combined effect of tumor necrosis factor-related apoptosis-inducing ligand and ionizing radiation in breast cancer therapy. Proc. Natl. Acad. Sci. USA.

[55] Hingtgen S., Ren X., Terwilliger E., Classon M., Weissleder R., Shah K. (2008). Targeting multiple pathways in gliomas with stem cell and viral delivered s-TRAILl and temozolomide. Mol. Cancer Ther..

[56] Keane M.M., Ettenberg S.A., Nau M.M., Russell E.K., Lipkowitz S. (1999). Chemotherapy augments TRAIL-induced apoptosis in breast cell lines. Cancer Res..

[57] Nimmanapalli R., Perkins C.L., Orlando M., O'Bryan E., Nguyen D., Bhalla K.N. (2001). Pretreatment with paclitaxel enhances Apo-2 ligand/tumor necrosis factor-related apoptosis-inducing ligand-induced apoptosis of prostate cancer cells by inducing Death Receptors 4 and 5 protein levels. Cancer Res..

[58] Mizutani Y., Yoshida O., Miki T., Bonavida B. (1999). Synergistic cytotoxicity and apoptosis by Apo-2 ligand and adriamycin against bladder cancer cells. Clin. Cancer Res..

[59] Matsubara H., Mizutani Y., Hongo F., Nakanishi H., Kimura Y., Ushijima S., Kawauchi A., Tamura T., Sakata T., Miki T. (2006). Gene therapy with TRAIL against renal cell carcinoma. Mol. Cancer Ther..

[60] Bonvini P., Zorzi E., Basso G., Rosolen A. (2007). Bortezomib-mediated 26s proteasome inhibition causes cell-cycle arrest and induces apoptosis in CD-30+ anaplastic large cell lymphoma. Leukemia.

[61] Williams S.A., McConkey D.J. (2003). The proteasome inhibitor bortezomib stabilizes a novel active form of p53 in human LnCAP-Pro5 prostate cancer cells. Cancer Res..

[62] Sayers T.J., Brooks A.D., Koh C.Y., Ma W., Seki N., Raziuddin A., Blazar B.R., Zhang X., Elliott P.J., Murphy W.J. (2003). The proteasome inhibitor PS-341 sensitizes neoplastic cells to TRAIL-mediated apoptosis by reducing levels of c-FLIP. Blood.

[63] Seki N., Toh U., Sayers T.J., Fujii T., Miyagi M., Akagi Y., Kusukawa J., Kage M., Shirouzu K., Yamana H. (2010). Bortezomib sensitizes human esophageal squamous cell carcinoma cells to TRAIL-mediated apoptosis via activation of both extrinsic and intrinsic apoptosis pathways. Mol. Cancer Ther..

[64] Brooks A.D., Ramirez T., Toh U., Onksen J., Elliott P.J., Murphy W.J., Sayers T.J. (2005). The proteasome inhibitor bortezomib (Velcade) sensitizes some human tumor cells to Apo2L/TRAIL-mediated apoptosis. Ann. N. Y. Acad. Sci..

[65] Rosato R.R., Almenara J.A., Coe S., Grant S. (2007). The multikinase inhibitor sorafenib potentiates TRAIL lethality in human leukemia cells in association with Mcl-1 and cFLIP_L_ down-regulation. Cancer Res..

[66] Llobet D., Eritja N., Yeramian A., Pallares J., Sorolla A., Domingo M., Santacana M., Gonzalez-Tallada F.J., Matias-Guiu X., Dolcet X. (2010). The multikinase inhibitor sorafenib induces apoptosis and sensitises endometrial cancer cells to TRAIL by different mechanisms. Eur. J. Cancer.

[67] Kim S.H., Ricci M.S., El-Deiry W.S. (2008). Mcl-1: A gateway to TRAIL sensitization. Cancer Res..

[68] Johnstone R.W. (2002). Histone-deacetylase inhibitors: Novel drugs for the treatment of cancer. Nat. Rev. Drug Discov..

[69] Norian L.A., Kucaba T.A., Earel J.K., Knutson T., Vanoosten R.L., Griffith T.S. (2009). Synergistic induction of apoptosis in primary B-CLL cells after treatment with recombinant tumor necrosis factor-related apoptosis-inducing ligand and histone deacetylase inhibitors. J. Oncol..

[70] Nakata S., Yoshida T., Horinaka M., Shiraishi T., Wakada M., Sakai T. (2004). Histone deacetylase inhibitors upregulate Death Receptor 5/TRAIL-R2 and sensitize apoptosis induced by TRAIL/Apo2-L in human malignant tumor cells. Oncogene.

[71] Fulda S., Debatin K.M. (2005). Resveratrol-mediated sensitisation to TRAIL-induced apoptosis depends on death receptor and mitochondrial signalling. Eur. J. Cancer.

[72] VanOosten R.L., Moore J.M., Karacay B., Griffith T.S. (2005). Histone deacetylase inhibitors modulate renal cell carcinoma sensitivity to TRAIL/Apo-2L-induced apoptosis by enhancing TRAIL-R2 expression. Cancer Biol. Ther..

[73] Vanoosten R.L., Moore J.M., Ludwig A.T., Griffith T.S. (2005). Depsipeptide (FR901228) enhances the cytotoxic activity of TRAIL by redistributing TRAIL receptor to membrane lipid rafts. Mol. Ther..

[74] Fulda S., Debatin K.M. (2005). HDAC inhibitors: Double edge sword for TRAIL cancer therapy?. Cancer Biol. Ther..

[75] Fulda S. (2008). Modulation of TRAIL-induced apoptosis by HDAC inhibitors. Curr. Cancer Drug Targets.

[76] Walther W., Stein U. (2000). Viral vectors for gene transfer: A review of their use in the treatment of human diseases. Drugs.

[77] Young L.S., Searle P.F., Onion D., Mautner V. (2006). Viral gene therapy strategies: From basic science to clinical application. J. Pathol..

[78] Lai C.M., Lai Y.K., Rakoczy P.E. (2002). Adenovirus and adeno-associated virus vectors. DNA Cell. Biol..

[79] Ginsberg H.S., Pereira H.G., Valentine R.C., Wilcox W.C. (1966). A proposed terminology for the adenovirus antigens and virion morphological subunits. Virology.

[80] Ballay A., Levrero M., Buendia M.A., Tiollais P., Perricaudet M. (1985). *In vitro* and *in vivo* synthesis of the hepatitis B virus surface antigen and of the receptor for polymerized human serum albumin from recombinant human adenoviruses. EMBO J..

[81] Karlsson S., Van Doren K., Schweiger S.G., Nienhuis A.W., Gluzman Y. (1986). Stable gene transfer and tissue-specific expression of a human globin gene using adenoviral vectors. EMBO J..

[82] Jones N., Shenk T. (1979). An adenovirus type 5 early gene function regulates expression of other early viral genes. Proc. Natl. Acad. Sci. USA.

[83] Kelly T.J., Lewis A.M. (1973). Use of nondefective adenovirus-simian virus 40 hybrids for mapping the simian virus 40 genome. J. Virol..

[84] Yang Y., Nunes F.A., Berencsi K., Gonczol E., Engelhardt J.F., Wilson J.M. (1994). Inactivation of E2a in recombinant adenoviruses improves the prospect for gene therapy in cystic fibrosis. Nat. Genet..

[85] Bergelson J.M., Cunningham J.A., Droguett G., Kurt-Jones E.A., Krithivas A., Hong J.S., Horwitz M.S., Crowell R.L., Finberg R.W. (1997). Isolation of a common receptor for coxsackie B viruses and adenoviruses 2 and 5. Science.

[86] Bergelson J.M., Krithivas A., Celi L., Droguett G., Horwitz M.S., Wickham T., Crowell R.L., Finberg R.W. (1998). The murine CAR homolog is a receptor for coxsackie B viruses and adenoviruses. J. Virol..

[87] Wickham T.J., Mathias P., Cheresh D.A., Nemerow G.R. (1993). Integrins alpha v beta 3 and alpha v beta 5 promote adenovirus internalization but not virus attachment. Cell.

[88] Hemmi S., Geertsen R., Mezzacasa A., Peter I., Dummer R. (1998). The presence of human coxsackievirus and adenovirus receptor is associated with efficient adenovirus-mediated transgene expression in human melanoma cell cultures. Hum. Gene Ther..

[89] Pearson A.S., Koch P.E., Atkinson N., Xiong M., Finberg R.W., Roth J.A., Fang B. (1999). Factors limiting adenovirus-mediated gene transfer into human lung and pancreatic cancer cell lines. Clin. Cancer Res..

[90] Jacob D., Davis J., Zhu H., Zhang L., Teraishi F., Wu S., Marini F.C., Fang B. (2004). Suppressing orthotopic pancreatic tumor growth with a fiber-modified adenovector expressing the TRAIL gene from the human telomerase reverse transcriptase promoter. Clin. Cancer Res..

[91] Jacob D., Bahra M., Schumacher G., Neuhaus P., Fang B. (2004). Gene therapy in colon cancer cells with a fiber-modified adenovector expressing the TRAIL gene driven by the hTERT promoter. Anticancer Res..

[92] Jacob D., Schumacher G., Bahra M., Davis J., Zhu H.B., Zhang L.D., Teraishi F., Neuhaus P., Fang B.L. (2005). Fiber-modified adenoviral vector expressing the tumor necrosis factor-related apoptosis-inducing ligand gene from the human telomerase reverse transcriptase promoter induces apoptosis in human hepatocellular carcinoma cells. World J. Gastroenterol..

[93] Mizuguchi H., Hayakawa T. (2002). Adenovirus vectors containing chimeric type 5 and type 35 fiber proteins exhibit altered and expanded tropism and increase the size limit of foreign genes. Gene.

[94] Wohlfahrt M.E., Beard B.C., Lieber A., Kiem H.P. (2007). A capsid-modified, conditionally replicating oncolytic adenovirus vector expressing TRAIL leads to enhanced cancer cell killing in human glioblastoma models. Cancer Res..

[95] Wang Y.G., Wang J.H., Zhang Y.H., Gu Q., Liu X.Y. (2004). Antitumor effect of a novel adeno-associated virus vector targeting to telomerase activity in tumor cells. Acta Biochim. Biophys. Sin. (Shanghai).

[96] Jimenez J.A., Li X., Zhang Y.P., Bae K.H., Mohammadi Y., Pandya P., Kao C., Gardner T.A. (2010). Antitumor activity of Ad-IU2, a prostate-specific replication-competent adenovirus encoding the apoptosis inducer, TRAIL. Cancer Gene Ther..

[97] Griffith T.S., Anderson R.D., Davidson B.L., Williams R.D., Ratliff T.L. (2000). Adenoviral-mediated transfer of the TNF-related apoptosis-inducing ligand/Apo-2 ligand gene induces tumor cell apoptosis. J. Immunol..

[98] Griffith T.S., Broghammer E.L. (2001). Suppression of tumor growth following intralesional therapy with TRAIL recombinant adenovirus. Mol. Ther..

[99] Armeanu S., Lauer U.M., Smirnow I., Schenk M., Weiss T.S., Gregor M., Bitzer M. (2003). Adenoviral gene transfer of tumor necrosis factor-related apoptosis-inducing ligand overcomes an impaired response of hepatoma cells but causes severe apoptosis in primary human hepatocytes. Cancer Res..

[100] VanOosten R.L., Griffith T.S. (2007). Activation of tumor-specific CD8+ T cells after intratumoral Ad5-TRAIL/CpG oligodeoxynucleotide combination therapy. Cancer Res..

[101] Berns K.I., Giraud C. (1996). Biology of adeno-associated virus. Curr. Top. Microbiol. Immunol..

[102] Lu Y. (2004). Recombinant adeno-associated virus as delivery vector for gene therapy--a review. Stem Cells Dev..

[103] Kwon I., Schaffer D.V. (2008). Designer gene delivery vectors: Molecular engineering and evolution of adeno-associated viral vectors for enhanced gene transfer. Pharm. Res..

[104] Mohr A., Henderson G., Dudus L., Herr I., Kuerschner T., Debatin K.M., Weiher H., Fisher K.J., Zwacka R.M. (2004). AAV-encoded expression of TRAIL in experimental human colorectal cancer leads to tumor regression. Gene Ther..

[105] Yoo J., Choi S., Hwang K.S., Cho W.K., Jung C.R., Kwon S.T., Im D.S. (2006). Adeno-associated virus-mediated gene transfer of a secreted form of TRAIL inhibits tumor growth and occurrence in an experimental tumor model. J. Gene Med..

[106] Wu Z., Asokan A., Samulski R.J. (2006). Adeno-associated virus serotypes: Vector toolkit for human gene therapy. Mol. Ther..

[107] Shi J., Zheng D., Liu Y., Sham M.H., Tam P., Farzaneh F., Xu R. (2005). Overexpression of soluble TRAIL induces apoptosis in human lung adenocarcinoma and inhibits growth of tumor xenografts in nude mice. Cancer Res..

[108] Bremer E., de Bruyn M., Samplonius D.F., Bijma T., ten Cate B., de Leij L.F., Helfrich W. (2008). Targeted delivery of a designed sTRAIL mutant results in superior apoptotic activity towards EGFR-positive tumor cells. J. Mol. Med..

[109] Wang Y., Huang F., Cai H., Zhong S., Liu X., Tan W.S. (2008). Potent antitumor effect of TRAIL mediated by a novel adeno-associated viral vector targeting to telomerase activity for human hepatocellular carcinoma. J. Gene Med..

[110] Zhang Y., Qu Z.H., Cui M., Guo C., Zhang X.M., Ma C.H., Sun W.S. (2009). Combined endostatin and TRAIL gene transfer suppresses human hepatocellular carcinoma growth and angiogenesis in nude mice. Cancer Biol. Ther..

[111] Wang Y., Ma L., Wang S., Bao Y., Ni C., Guan N., Zhao J., Fan X. (2009). Assessment of CAR- or CD46-dependent adenoviral vector-mediated TRAIL gene therapy in clinical adenocarcinoma lung cancer cells. Oncology.

[112] Ma H., Liu Y., Liu S., Xu R., Zheng D. (2005). Oral adeno-associated virus-sTRAIL gene therapy suppresses human hepatocellular carcinoma growth in mice. Hepatology.

[113] Wenger T., Mattern J., Haas T.L., Sprick M.R., Walczak H., Debatin K.M., Buchler M.W., Herr I. (2007). Apoptosis mediated by lentiviral TRAIL transfer involves transduction-dependent and - independent effects. Cancer Gene Ther..

[114] Mangipudi S.S., Canine B.F., Wang Y., Hatefi A. (2009). Development of a genetically engineered biomimetic vector for targeted gene transfer to breast cancer cells. Mol. Pharm..

